# Two new Oribatid mites from Costa Rica, *Mixacarus turialbaiensis* sp. n. and *Paulianacarus costaricensis* sp. n. (Acari, Oribatida, Lohmanniidae)

**DOI:** 10.3897/zookeys.680.13213

**Published:** 2017-06-14

**Authors:** Nestor Fernandez, Pieter Theron, Sergio Leiva, Louwrens Tiedt

**Affiliations:** 1 National Council of Scientific and Technological Research, Argentina (CONICET). Subtropical Biological Institute (IBS). Evolutionary Genetic Laboratory FCEQyN, Misiones National University. Felix de Azara 1552, 6º, (3300) Posadas Misiones Argentina; 2 Research Unit for Environmental Sciences and Management, North-West University, Potchefstroom Campus, 2520, South Africa; 3 Fellowship, National Institute Agricultural Technology (INTA). Experimental Rural Agency, Aimogasta. La Rioja, Argentina; 4 Laboratory for Electron Microscopy, North-West University, Potchefstroom Campus, 2520, South Africa

**Keywords:** Acari, Oribatida, Lohmanniidae, Costa Rica, *Mixacarus turialbaiensis* sp. n., *Paulianacarus costaricensis* sp. n.

## Abstract

In this paper we describe two new species belonging to the family Lohmanniidae: *Mixacarus turialbaiensis* **sp. n.** and *Paulianacarus costaricensis* **sp. n.** from Costa Rica.

## Introduction

Approximately three years ago, the authors commenced the study of materials housed at the Museum d’Histoire Naturelles de Genève (MHNG), which was collected from the Turrialba forest in Costa Rica. In this initial paper, we describe two new species belonging to genera *Mixacarus* and *Paulianacarus* of the family Lohmanniidae. The taxonomy of the first species, *Mixacarus turialbaiensis* sp. n., was problematic as taxonomically important characters of related species were not adequately described in most prior studies. For the second species, *Paulianacarus costaricensis* sp. n., the situation is similar, but with seemingly misinterpreted original descriptions an aggravating factor.

## Material and methods

Specimens studied by means of light microscopy were macerated in lactic acid, and observed in the same medium using the open-mount technique (cavity slide and cover slip) as described by Grandjean (1949), Krantz and Walter (2009). Drawings were made using a Zeizz GFL (Germany) compound microscope equipped with a drawing tube. Specimens preserved in ethanol, studied under Scanning Electron Microscope (SEM), were carefully rinsed by sucking them several times into a Pasteur pipette, after which they were transferred to buffered glutaraldehyde (2,5%) in Sörensen phosphate buffer: pH 7,4; 0,1 m for two hours. After postfixation for 2hr. in buffered 2% OsO4 solution and being rinsed in buffer solution; all specimens were dehydrated in a series of graded ethanol and dried in a critical point apparatus. After mounting on Al-stubs with double sided sticky tape, specimens were gold coated in a sputter apparatus (Alberti and Fernandez 1988, 1990a, 1990b; Alberti et al. 1991, 1997, 2007; Fernandez et al. 1991). For SEM observations, a FEI-Quanta Feg 250 Scanning Electron Microscope; with 10 Kv and working distant (WD) variable was used.

Measurements taken: total length (tip of rostrum to posterior edge of notogaster); width (widest part of notogaster) in micrometers (μm). Setal formulae of the legs include the number of solenidia (in parentheses); tarsal setal formulae include the famulus (ε).

### Morphological terminology and abbreviations

Morphological terms and abbreviations used are those developed by Grandjean (1928–1974) (cf. Travé and Vachon, 1975; Norton and Behan-Pelletier (in Krantzand Walter 2009);); Norton and Behan-Pelletier (2009); Fernandez et al. (2013a–c).

**Institutions**

**MHNG** (Muséum d‘Histoire Naturelles, Genève, Switzerland).

## New taxa descriptions

### Family Lohmanniidae Berlese, 1916

#### Genus *Mixacarus* Balogh, 1958

##### Mixacarus turialbaiensis sp. n.

Taxon classificationAnimaliaarcoptiformesLohmanniidae

http://zoobank.org/BE1D6634-752A-4E97-8E51-128B3E96F96E

Figs 1–8 , 9–11 , 12–15 , 16–18 , 19–23 , 24–28 ; Table 1

###### Etymology.

The specific epithet is dedicated to the Turrialba forest of Costa Rica, where the specimens were collected.

**Table 1.** *Mixacarus turrialbai* sp. n.: setae and solenidia.

	Femur	Genu	Tibia	Tarsus	Claw
**Leg I**					
setae	*l”,d,v*	*l”,d*	*l”,v*	*(p),(u),(a),σ,(it),(tc),(ft),(pv),e*	1
solenidia		σ, σ	φ	ω_1_ , ω_2_	
**Leg II**					
setae	*la,lp,vb,v*	*d,l*”,	*d,l”,v*	*(p),(u),(a),σ,(tc),(ft),(pv)*	1
solenidia		σ	φ	ω	
**Leg III**					
setae	*l’,v*	*d,l’,v*	*d,l’,v*	*(p),(u),(a),σ,(tc),(ft),(pv)*	1
solenidia		σ	φ		
**Leg IV**					
setae	*d,l’,v*	*d,l*’	*d,l’v*	*(p),(u),(a),σ,(tc),(ft),(pv)*	1
solenidia		σ			

###### Type material.

**Holotype.** Label details: “CCR 0978 Tu 11 Costa Rica Turrialba forêt naturelle du catie alt. 560 m. Triage d’humus cote est surface nid d’*Atta* au pied de *Castilla elastica* 1.IX. 1978. LEG P.WERNER 10.140744, alt. 120 m” conserved in 70% ethanol, deposited in MHNG.

**Paratypes.** same data, 2 ♀♀ deposited in MHNG; preserved in 70% ethanol.

###### Diagnosis

(adult female). Setae *ro* inserted anteriorly on transversal cuticular ridge; *le*, *in* setae erect; setae *ro*, *le*, *in* more or less similar length. Several ribbon-like bands near *ro*, *le*, *exa*, *exp* setae; sensillus pectinate (6–9 pectines); clearly visible superior cornea of naso (*CSO)*.

Sixteen pairs of setae: *c_1_, c_2_, c_3_, d_1_, d_2_, d_3_, e_1_, e_2_, f_1_, f_2_, h_1_ , h_2_, h_3_, p_1_, p_2_, p_3_*; eight transversal bands: *S2, S3, S4; S5, S6, S7, S8, S9*. Bands *S2, S6, S8, S9* cross medial notogastral plane transversally; *S3, S4, S5, S7* not crossing medial notogastral plane. Five pairs of lyrifissures: *ia, ip, ips, im, ih*.

Adoral setae: *or*_1_ spoon-shaped, largest; *or*_2_ elongate, tip beak-shaped; *or*_3_ large, rounded apex. Epimeral setal formula 3–1–3–(3–4), epimere IV with either three or four pairs of setae; genital plate undivided, rounded elevated central zone bearing nine or ten pairs of setae; six or seven pairs of simple setae aligned paraxially,

###### Description

**(female).** *Measurements*. 525 (485–560) × 233 (224–245) (ten specimens measured).

**Figures 1–8.**
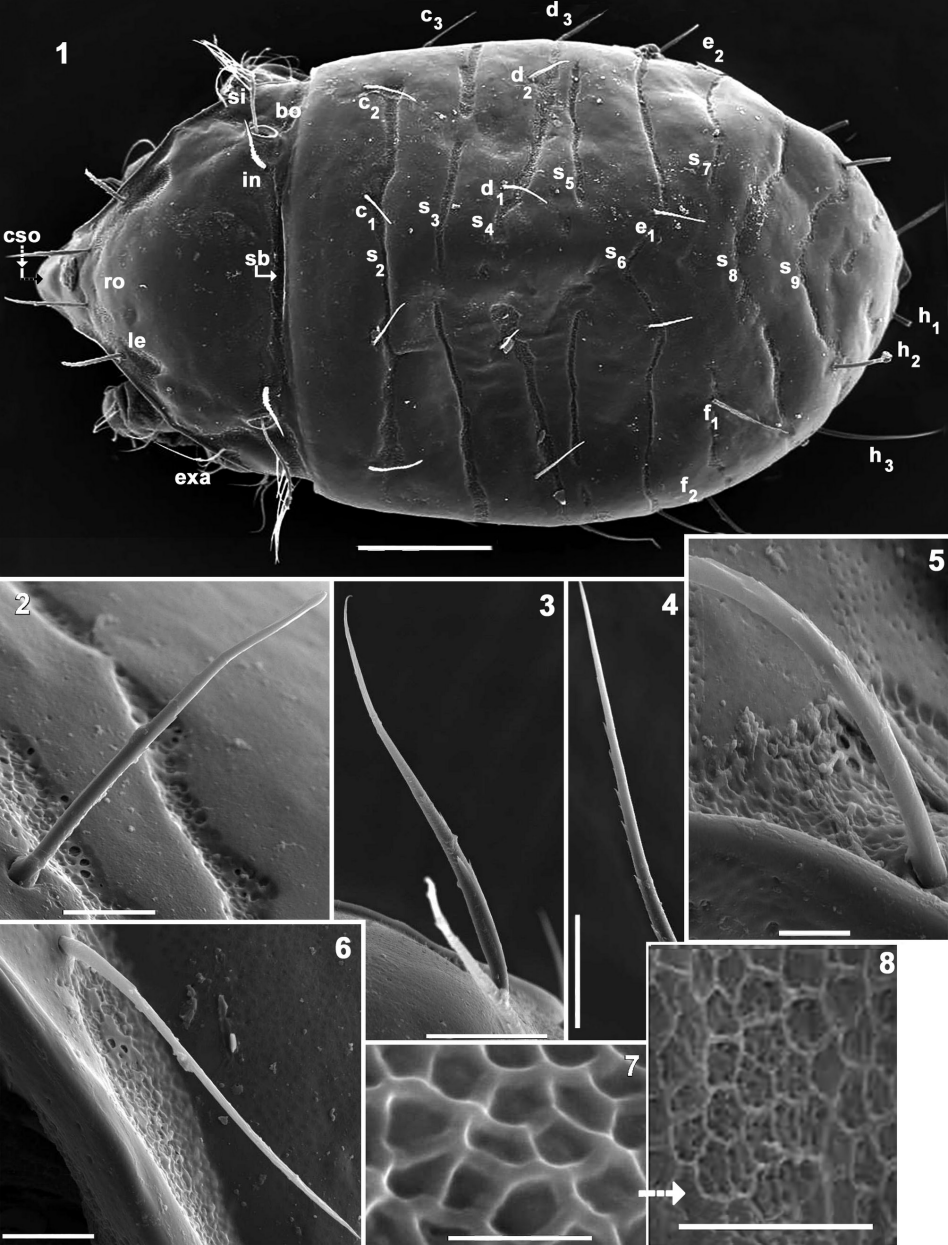
*Mixacarus turialbaiensis* sp. n. Adult with cerotegumental layer. SEM. **1** dorsal view **2** notogastral setae *d_3_* **3** *ro* setae **4** *in* setae **5** *exa* setae **6** *exp* setae **7** detail of cuticular microsculpture **8** polyhedral microsculpture from porose area. Abbreviations: See Material and methods. Scale bars: **1** = 100 μm; **2** = 20 μm; **3** = 10 μm; **4** = 20 μm; **5** = 5 μm; **6** = 10 μm; **7** = 2 μm; **8** = 5 μm.

**Figures 9–11.**
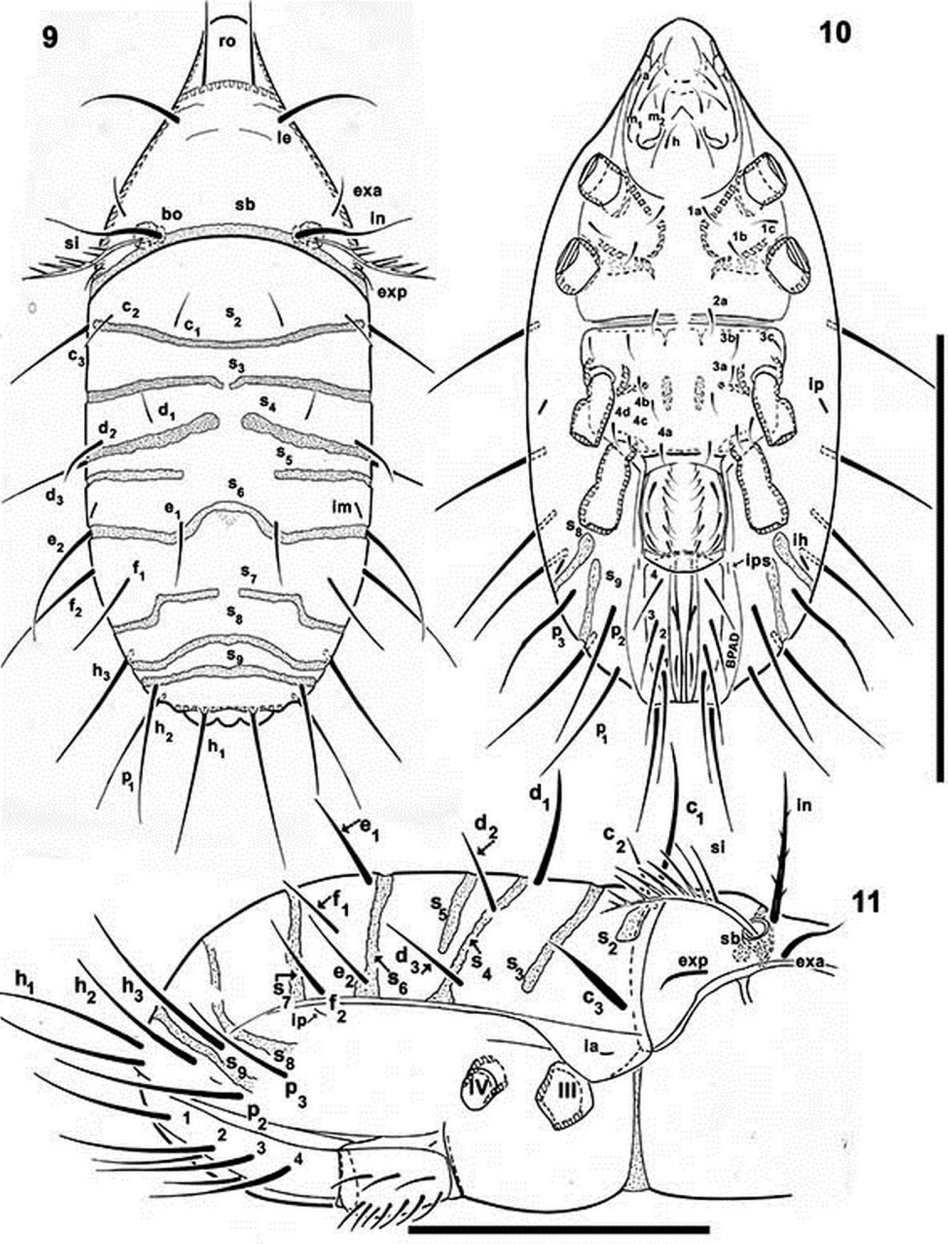
*Mixacarus turialbaiensis* sp. n. Adult, optical microscopy. **9** dorsal view **10** ventral view **11** lateral view. Abbreviations: See Material and methods. Scale bars: 9, 10 = 300 μm; 11 = 200 μm.

**Figures 12–15.**
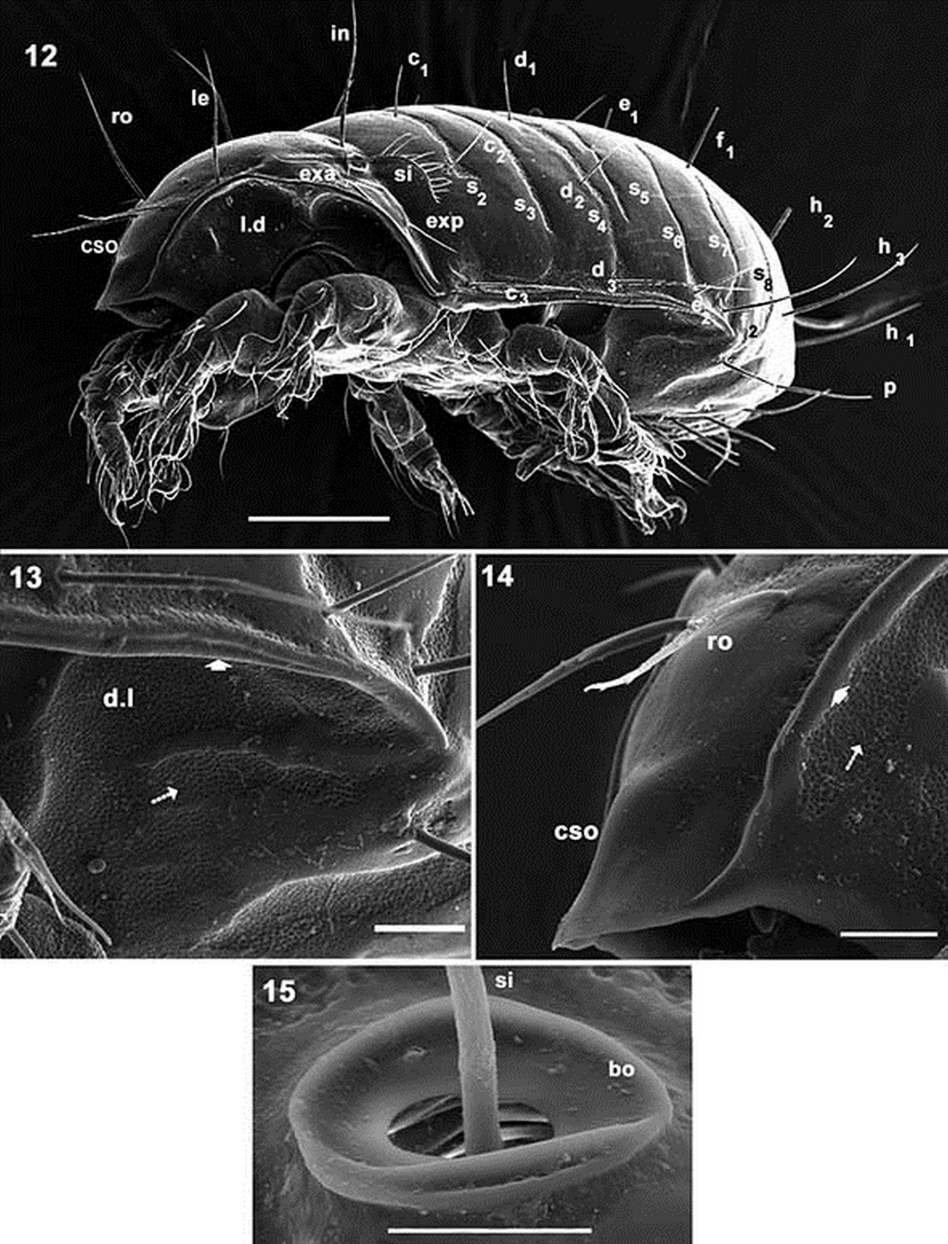
*Mixacarus turialbaiensis* sp. n. Adult with cerotegumental layer. SEM. **12** lateral view **13** microsculpture lateral zone **14** anterior prodorsal zone **15** bothridial zone. Abbreviations: See Material and methods. Scale bars: **12** = 100 μm; **13** = 20 μm., **14** μm = 20 μm; **15** = 10 μm.

*Shape.* Oval (Figures 1, 9, 10, 12).

*Colour.* Yellow to light brown; slightly shiny when observed in reflected light.

*Cerotegument.* Almost nonexistent; or disappeared during extensive period of conservation in ethanol.

*Integument.* Smooth: prodorsum, notogaster, ventral region (Figures 1, 12); depressed areas of variable size with polyhedral microsculpture (Figure 7): *sb* (ribbon-like prodorsal bands) (Figure 1); lateral prodorsal zone (Figures 5, 6); zone of *l.d* (Figures 8, 13, 14); notogastral band *S2, S3, S4, S5, S6, S7, S8, S9* (Figures 1, 2, 9, 11, 12, 19); notogastral marginal zone (Figures 12, 19); subcapitular zone around setae *h*, *m_1_*, *m_2_*, *a* (Figure 20); epimeral zone (Figures 16, 17, 21, 23); anogenital zone (Figure 18); ventral zone external to anogenital zone (Figures 16, 18); legs (Figures 24–28).

*Setation* (legs not included). Two types: *simple, smooth*: genital, anal (Figures 1, 2, 18); *simple, barbed*: prodorsum, notogaster, epimeral, subcapitular (Figures 3, 4, 5, 14, 21, 23). Barbs are small, difficult to observe.

*Prodorsum*. Shape: triangular, rounded apex in dorsal view (Figures 1, 9); triangular in lateral view (Figures 12, 14). Rostrum broadly rounded (Figures 1, 9); elevated chitinous ridge present on either side of prodorsal area, externally to *exa*, *exp*, *le* setae, derived from margins of leg depressions (Figures 12, 14); *ro* setae inserted anteriorly on transversal cuticular ridge, generally directing forward (Figures 1, 9, 12); *le*, *in* setae erect (Figure 12); setae *ro*, *le*, *in* more or less similar length. Several ribbon-like bands near *ro*, *le*, *exa*, *exp* setae, extending laterally to elevated lateral ridge (Figures 5, 6, 12, 13). *Bo* rounded, slightly elevated from the cuticular surface (Figure 15), laterally tilted (Figures 1, 9, 12). Sensillus pectinate (6–9 pectines) (Figures 9, 11, 12). Postbothridial transverse band *sb* clearly discernible, situated posterior to *bo* and *in* setae (Figures 1, 9, 11). On anterior zone near apex, in front of *ro* setal insertion and between cuticular elevations of *l.d*, *CSO* clearly visible (Figures 1, 12, 14).

*Notogaster.* Sixteen pairs of primary notogastral setae: *c_1_, c_2_, c_3_, d_1_, d_2_, d_3_, e_1_, e_2_, f_1_, f_2_, h_1_, h_2_, h_3_, p_1_, p_2_, p_3_* clearly discernible (Figures 1, 9, 11, 12). Nine transversal bands: *S2, S3, S4, S5, S6, S7, S8, S9* (Figures 1, 9, 11, 12); *S2* crossing transverse medial notogastral plane, exceeding slightly beyond *c_2_* setae, terminating near *c_3_* in a large rectilinear tip (Figures 11, 12); *S3* situated behind *c* setal alignment and in front of *d* setal alignment, not crossing medial notogastral plane; laterally stopping above *c_3_*, *d_3_* setal insertion level (Figures 11, 12); S4 observed anterior to *d* setal alignment, not crossing medial notogastral plane, running obliquely, exceeding *d*_1_ setal insertion level, terminating in rounded end (Figures 1, 9); S4 extending to unsclerotized lateral longitudinal line (Figures 11, 12); S5 thin (Figures 1, 9), not crossing medial notogastral plane, laterally terminating before *d*_3_ setal insertion level (Figures 11, 12); S6 situated behind *e_1_*, crossing medial notogastral plane (Figures 1, 9), laterally reaching unsclerotized lateral longitudinal line (Figures 1, 9, 11, 12); S7 situated behind *f*_1_ setal insertion, not crossing medial notogastral plane, extending to unsclerotized lateral longitudinal line (Figures 1, 9, 11, 12); S8, S9 crossing medial notogastral plane and unsclerotized lateral longitudinal line (Figure 11).

Five pairs of lyrifissures present: *ia*, *ip* situated below the unsclerotized lateral longitudinal line (see Lateral region); *ips* situated on the adanal fold band (BPDA) (Figures 9, 10, 11); *im* near *e_2_* setae and *ih* behind *h*_3_.

*Lateral region*. Prodorsal margin present on either side of cavities housing legs I-IV when retracted. Anterior notogastral zone presenting conspicuous tectum and clearly defined unsclerotized lateral longitudinal line, terminating almost posterior to level of *ip* lyrifissure and delimiting unpaired dorsal notaspis and pleuraspis (paired narrow lateral zones) (Figure 11). In posterior notogastral zone, when unsclerotized line does not exist, notaspis and pleuraspis not delimited (Figure 11). Each pleuraspis presenting an anterior rounded lobe between legs II and III, where lyrifissure *ia* is observed. Posteriorly, at level of *d_3_* and *e_2_* setae, well delimited edges form canopies over cavities in which legs III and IV are housed when retracted, with a protruding angle between them.

*Ventral region*. Anterior zone of subcapitulum more or less triangular, posterior zone ovoid. Four pairs of subcapitular setae (Figure 10) *h*, *m_1_, m_2_, a*. Characteristic adoral setae: *or_1_* largest, spoon shaped; *or_2_* elongate, terminating in beak-shape; *or_3_* large, rounded apex (Figure 20).

**Figures 16–18.**
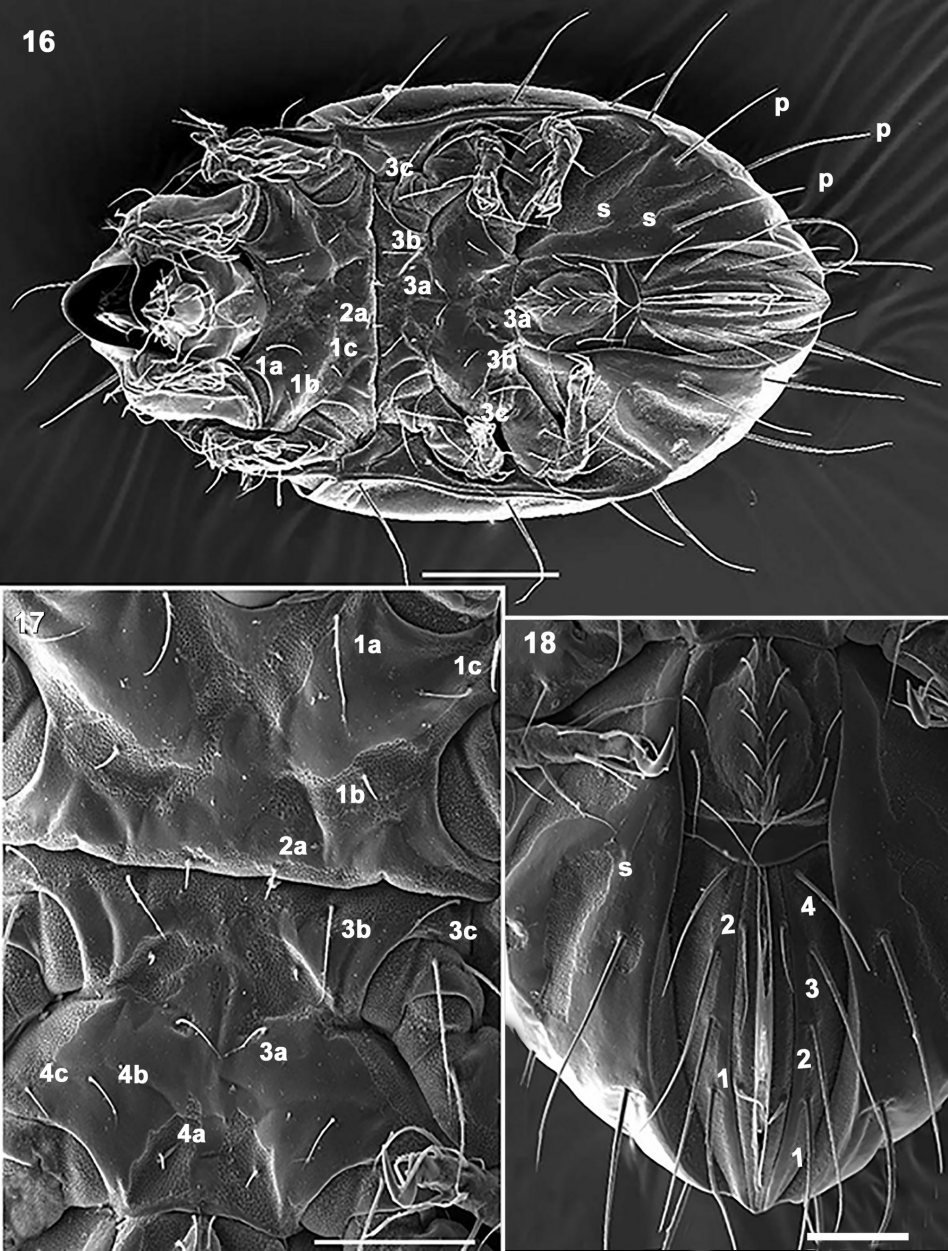
*Mixacarus turialbaiensis* sp. n. Adult with cerotegumental layer. SEM. **16** ventral zone **17** Epimeral zone **18** anogenital region. Abbreviations: See Material and methods. Scale bars: **16**=100 μm; **17, 18**= 50 μm.

Coxisternal region divided into two parts by ventrosejugal groove (Figures 10, 16, 17). Apodemes short and clearly visible; epimeral setal formulae 3-1-3-(3-4), epimere IV with three or four pairs of setae; all setae similarly shaped, but vary in length (Figures 21, 22, 23). Genital plate undivided, elevated central zone rounded with ten pairs of setae, sometimes with only nine pairs; (Figures 10, 16, 18); six or seven simple setae aligned paraxially, and three antiaxially. Preanal plate more or less triangular, rounded central zone.

Anal and adanal plates with four pairs of adanal and two pairs anal setae (Figures 16, 18). Band BPAD clearly visible in specimens immersed in lactic acid for lengthy period; lyrifissure *ips* present near margin of this band (Figure 10).

*Legs.* Two types of femora can be distinguished. Femora of legs I and II displaying large ventral blade (Figures 24, 25), femora of legs III and IV lacking ventral blade (Figures 26, 27).

Setal formulae I (0–3–2–2–16–1) (2–1–2); II (0–4–2–3–13–1) (1–1–1); III (2–3–2–2–13–1) (1–1–0); IV (2–3–2-3–13–1(1–0–0). See Table 1.

**Figures 19–23.**
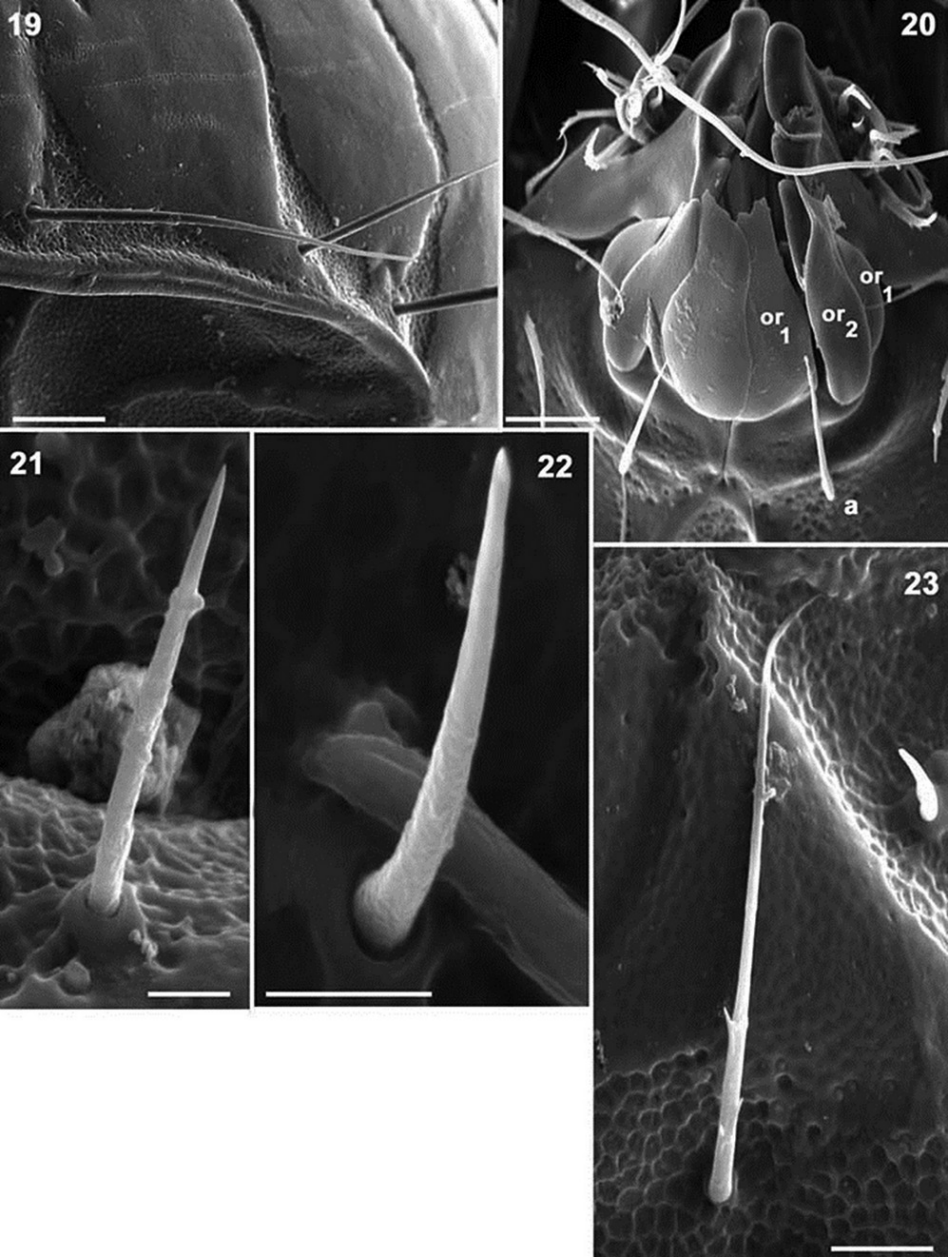
*Mixacarus turialbaiensis* sp. n. Adult with cerotegumental layer. SEM. **19** lateral notogastral zone **20** adoral setae **21** epimeral zone, *2a* setae **22** epimeral zone, *3a* setae **23** epimeral zone, *3b* setae. Abbreviations: See Material and methods. Scale bars: **19** = 20 μm; **20** = 10 μm; **21, 22** = 2 μm; **23** = 5 μm.

**Figures 24–28.**
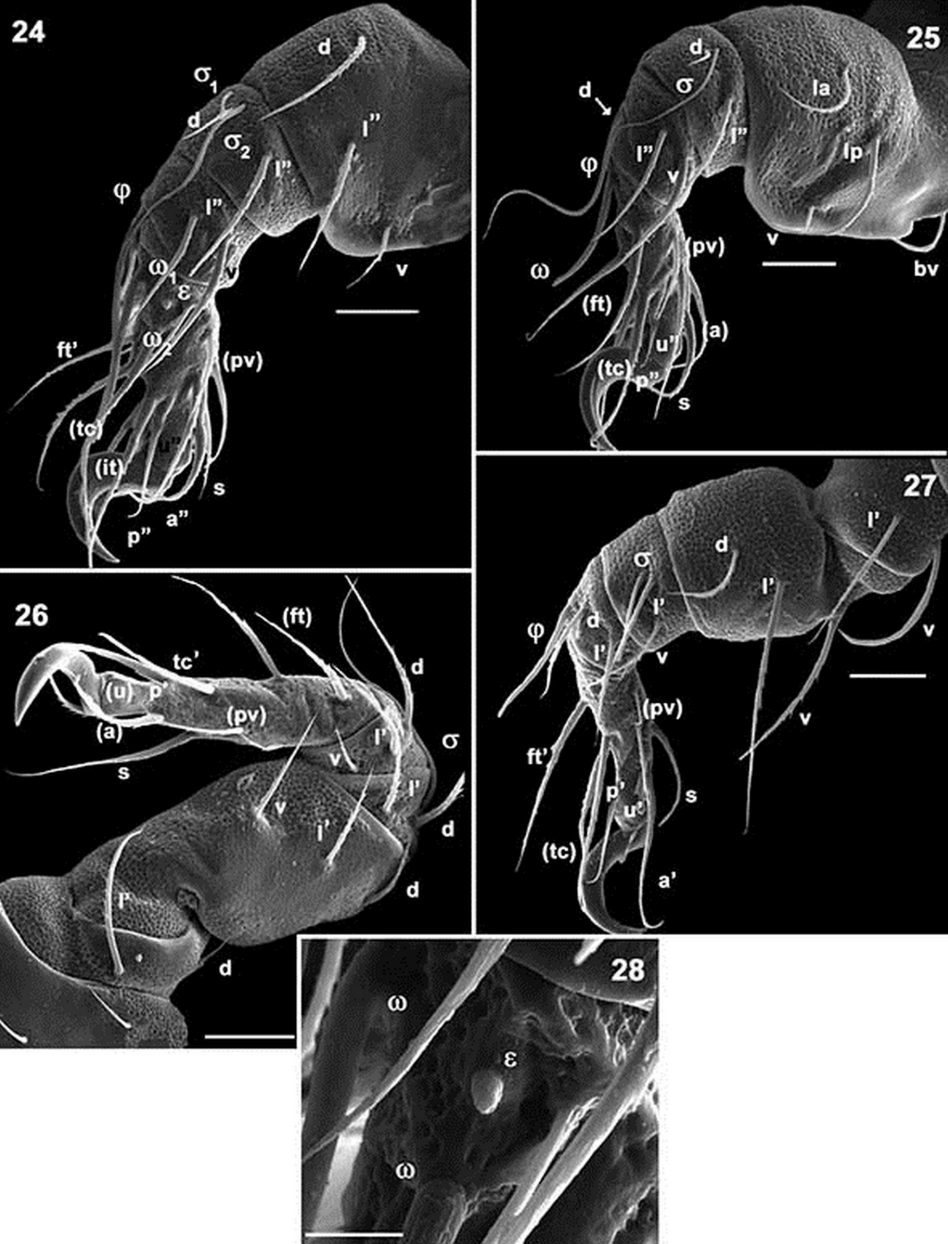
*Mixacarus turialbaiensis* sp. n. Adult with cerotegumental layer. SEM. **24** leg I antiaxial view **25** leg II antiaxial view **26** leg IV antiaxial view **27** leg III antiaxial view **28** tarsus I, famulus zone. Abbreviations: See Material and methods. Scale bar: **23** = 50 μm; **24** = 20 μm; **25** = 20 μm; **26** = 20 μm; **27** = 20 μm; **28** = 5 μm.

#### Genus *Paulianacarus* Balogh, 1960

##### Paulianacarus costaricensis sp. n.

Taxon classificationAnimaliaarcoptiformesLohmanniidae

http://zoobank.org/662FF0B7-A77E-441D-90D2-2720B07FA833

Figs 29–30 , 31–32 , 33–35 , 36–41 , 42–47 , 48–51 , 52–55 ; Table 2

###### Etymology.

The specific epithet is dedicated to Costa Rica *costaricensis* (Latin = from Costa Rica), the country where the specimens were collected.

**Table 2.** *Paulianacarus costaricensis* sp. n.: setae and solenidia.

	Femur	Genu	Tibia	Tarsus	Claw
**Leg I**					
setae	*(l),d,v*”	*(l),d*	*(l),v*	*(p),(u),(a),σ,(it),(tc),(ft),(pv), ε*	1
solenidia		σ’, σ’’	φ	ω_1_ , ω_2_	
**Leg II**					
setae	*d,la”lp”,l’,vb, v*	*d,l”,xt*	*d,(l),v,xt*	*(p),(u),(a),σ,(tc),(ft),(pv)*	1
solenidia		σ	φ	ω	
**Leg III**					
setae	*l’,v*	*d,l’,v*	*d,l’,v*	*(p),(u),(a),σ,(tc),(ft),(pv)*	1
solenidia		σ	φ		
**Leg IV**					
setae	*d,l’,v*	*d l’,v*	*d,l*’	*(p),(u),(a),σ,(tc),(ft),(pv)*	1
solenidia		σ			

###### Type material.

**Holotype.** Label details: “♀ CR 0978 Tu 18a. Costa Rica Turrialba forêt naturelle du catie alt. 560 m. Racines d’epiphytes sur branche tombe 1 mois avant. 24. IX. 1978 LEG P.WERNER”. MHNG, preserved in 70% ethanol. **Paratypes**: same data and locality 2 ♀♀. MHNG, preserved in 70% ethanol.

###### Diagnosis.

*Prodorsum.* Triangular to slightly polyhedral; rostrum rectilinear; *ro* setae inserted far from rostrum; *si* pectinate (5-8 pectines). *Notogaster*. Sixteen pairs of setae: *c_1_, c_2_, c_3_, d_1_, d_2_, d_3_, e_1_, e_2_, f_1_, f_2_, h_1_, h_2_, h_3_, p_1_, p_2_, p_3_*. Cuticular surface with nine elevated transversal thickenings; 1-5 complete, crossing medial notogastral plane; 6-9 not crossing medial notogastral plane; elevated transversal thickening, nine transverse bands present; 3, 4, 7 smooth, others with promontories.

###### Description

(Adult female). *Measurements.* Length 960 (1100–890) ×535 (526–540) (three specimens).

**Figures 29–30.**
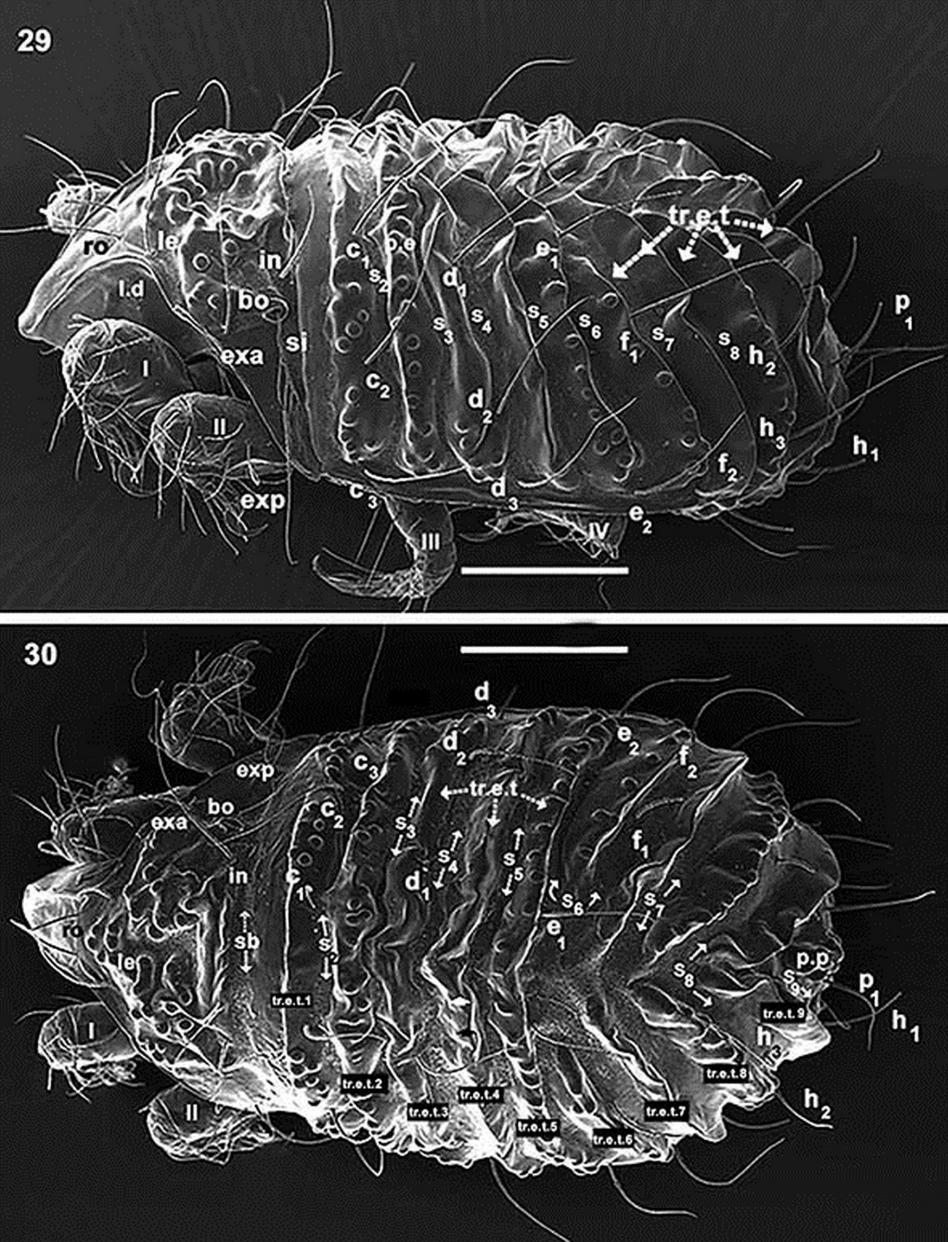
*Paulianacarus costaricensis* sp. n. Adult with cerotegumental layer. SEM. **29** lateral inclined view **30** dorsal with slight lateral tilt. Abbreviations: See Material and methods. Scale bars: **29, 30** = 200 μm.

**Figures 31–32.**
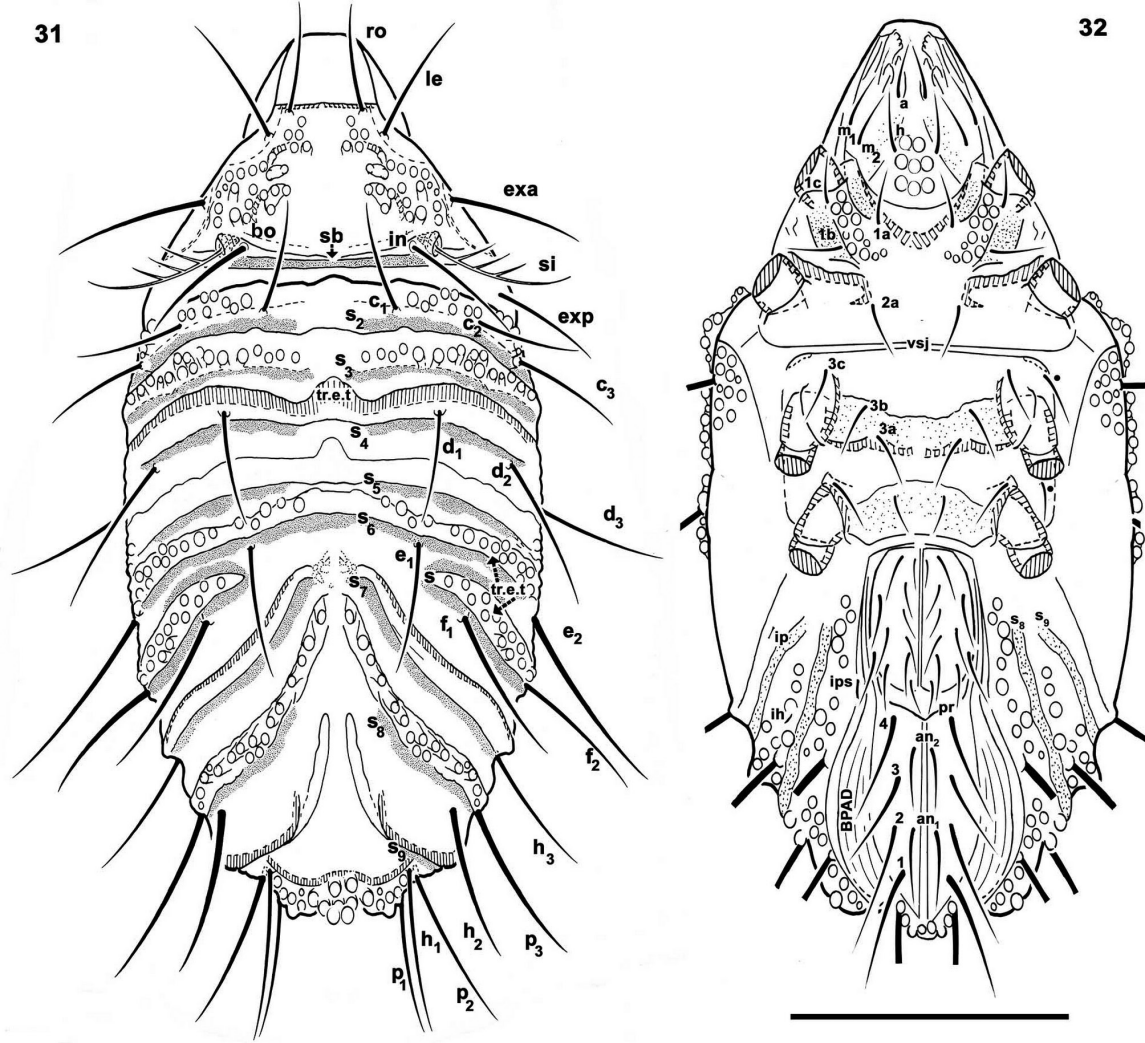
*Paulianacarus costaricensis* sp. n. Adult, optical microscopy**. 31** dorsal view **32** ventral view. Abbreviations: See Material and methods. Scale bar: **31**, **32** = 400 μm.

*Shape.* Elongate ovoid (Figures 29, 30, 31, 32).

*Colour.* Dark to light brown; slightly shiny when observed in reflected light.

*Cerotegument.* Nonexistent.

*Integument.* Complex microsculpture. Rounded promontories (Figures 29, 30, 38, 40, 41); elevated transversal thickening (*tr.e.t*) (Figures 29, 30, 31, 33); polyhedral microsculpture (0.7–0.8) (Figures 42, 43, 44, 45, 46 indicated by arrow) in depressed areas (Figure 46, under large magnification), this type of microsculpture observed on cuticular structures on various areas of body and legs (Figures 42, 43, 44, 45 indicated by u), principally on transverse bands.

*Setation* (legs not included). Two types: *simple, smooth*: prodorsum: *le*, *ro* length 163 (140–180); *exp, exa* length 153 (140–160); notogaster: 167 (140–180); epimeral (40–53); genital 53 (40–52); aggenital 59.5 (45–72); anal 74 (63–81); adanal 119 (100–136); subcapitular (*h*, *m*) 51.5 (50–54); *a* 39.5 (36–41) (Figures 49, 50); *simple barbate*: *in* setae 142 (130–150) (Figures 38, 41).

*Prodorsum.* Triangular to slightly polyhedral in dorsal view (Figures 30, 31); triangular in lateral view (Figures 29, 34).

**Figures 33–35.**
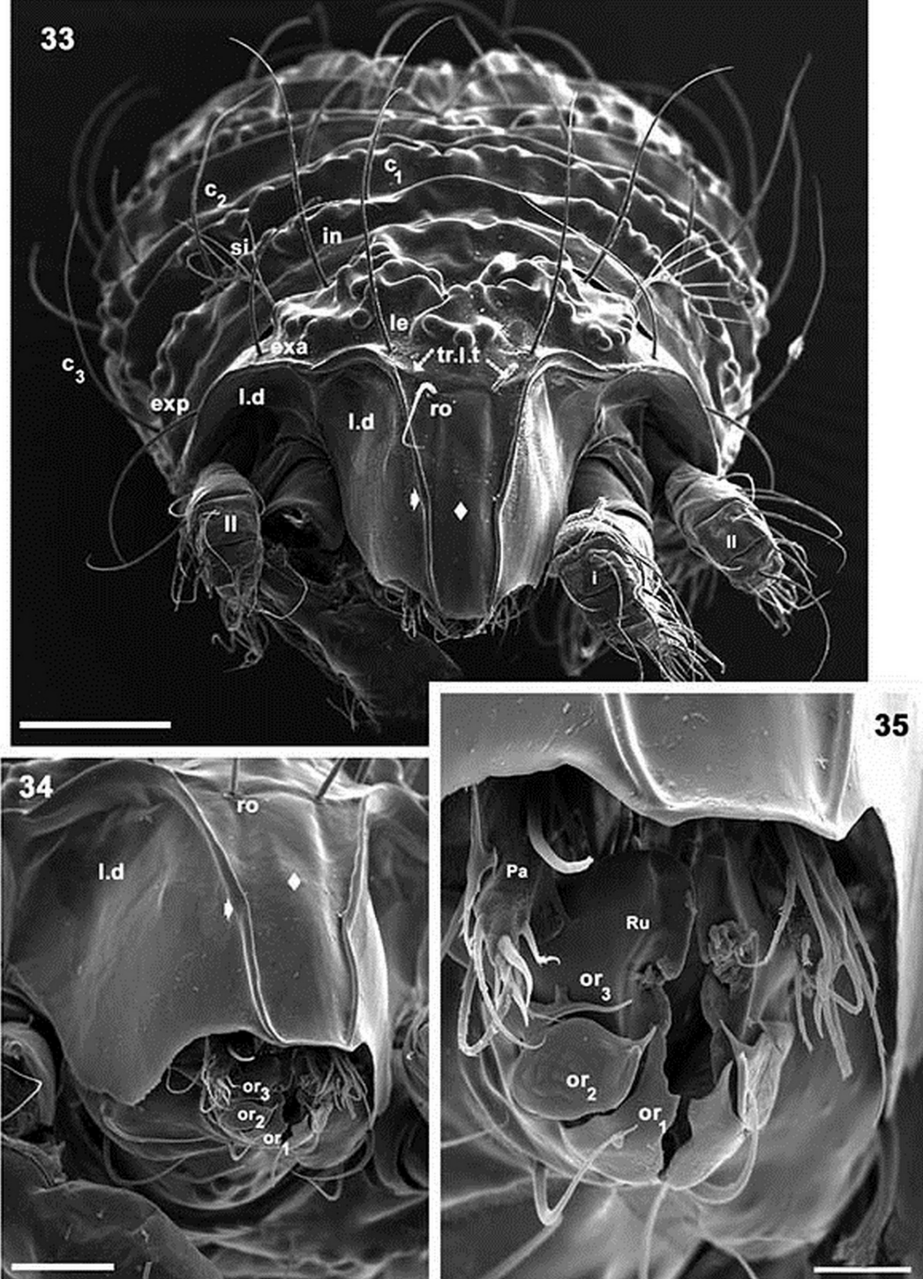
*Paulianacarus costaricensis* sp. n. Adult with cerotegumental layer. SEM. **33** frontal view **34** prodorsum, laterally inclined **35** apical zone, infracapitulum. Abbreviations: See Material and methods. Scale bars: **33** = 100 μm; **34** = 50 μm; **35** = 20 μm.

**Figures 36–41.**
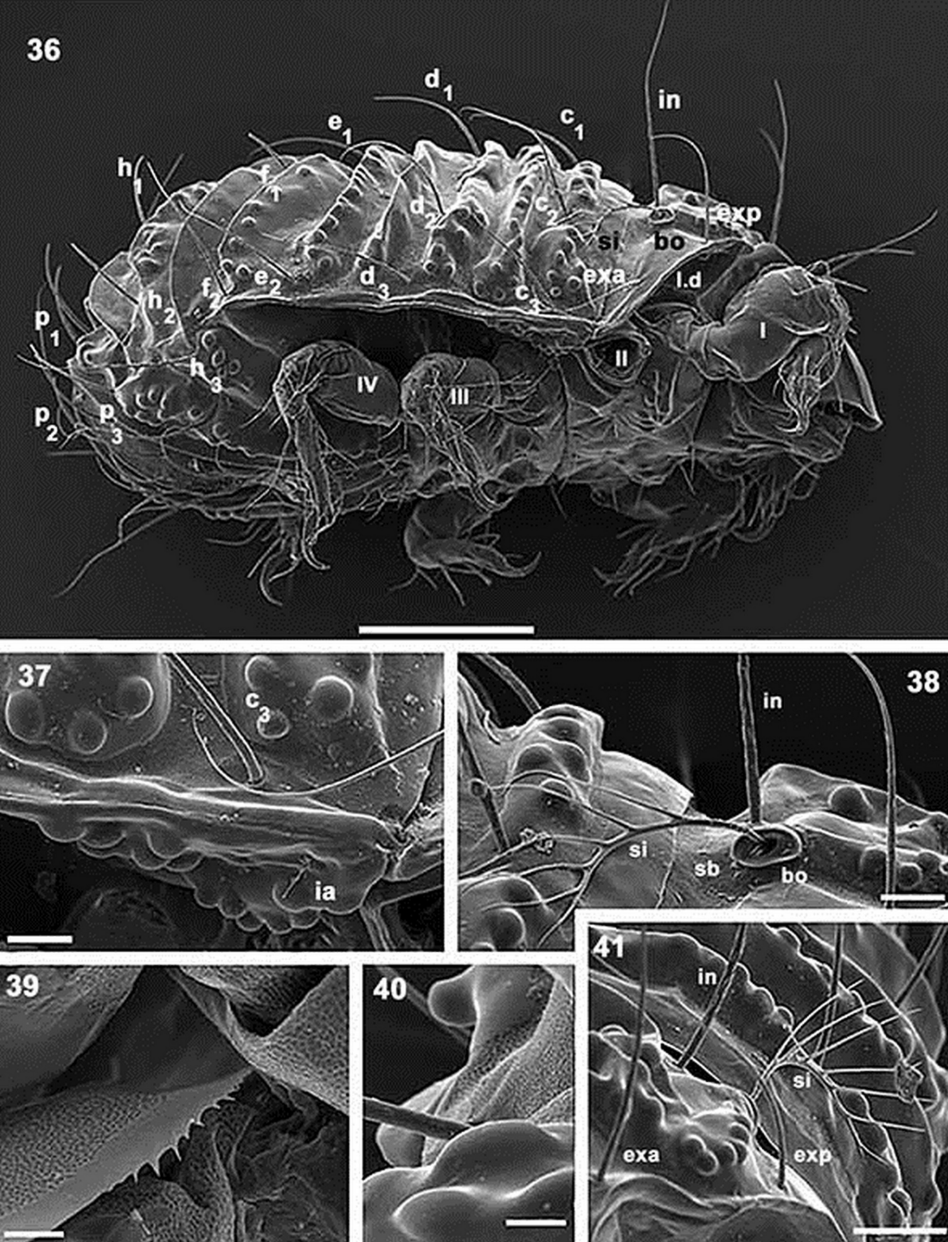
*Paulianacarus costaricensis* sp. n. Adult with cerotegumental layer. SEM. **36** lateral view **37** anterior lateral notogastral zone **38** bothridial zone **39** prodorsal marginal zone **40** promontories **41** lateral view, sensillus zone. Abbreviations: See Material and methods. Scale bars: **36** = 200 μm; **37** = 20 μm; **38** = 50 μm; **39** = 10 μm; **40** = 10 μm; **41** = 50 μm.

Rostrum rectilinear (Figures 33–35). Prodorsal margin dentate (Figures 34, 39). Depression housing legs *l.d* (for legs I and II) (Figure 36) clearly observed as laterally situated concave arc-shaped zone; *ro* setae inserted far from rostrum, in some instances situated slightly anterior to *tr.l.t* (transversal elevated thickening) (Figures 29, 30, 31); margins of *l.d* formed by elevated cuticular thickening (indicated by arrows ¿ Figures 33, 34). Medial prodorsal zone, situated between *sb* (transverse postbothridial band), transversal linear thickening (*tr.l.t*) and setae *exp*, *exa*, *le*, with prominent elevated round promontories (Figure 33); smooth polyhedral area situated between *l.d* elevated margins, *tr.l.t* and rostrum; with an interior rectangular zone (Figures 33, 34 indicated by s); *le* setal insertion anterior to *tr.l.t* (Figure 33), situated near l.d elevated margin (Figure 33, indicated by arrows ¿); *bo* cup-shaped, dorsally open (Figures 36, 38); *si* pectinate, with 5–8 large pectines (Figures 33, 38, 41); *in* setae inserted at level of *bo*, situated internally to *bo* and in front of *sb* (Figures 30, 31); *exa* and *exp* well visible, situated marginally on a smooth area (Figure 43); *sb* clearly discernible, situated behind *in* setal insertions (Figures 30, 31).

*Frontal view.* Rostrum rectilinear, situated in medial zone between *l.d* elevated cuticular thickening (Figures 33, 34, indicated by arrows ¿); prodorsal border at first concave and becoming convex towards the posterior; in boundary zone between concave and convex, a series of dentate projections (Figures 34, 39). Anterior subcapitular zone (Figures 32, 34, 35), adoral setae clearly visible: *or*_3_ sigmoid; *or*_2_ very complex, leaf-shaped in ventral view (Figure 35), in lateral view (Figure 46) resembling a bird’s head and beak; *or*_1_ very complex, resembling a leaf with edges eaten by a caterpillar (Figures 35, 46).

*Notogaster.* Sixteen pairs of notogastral setae: *c_1_, c_2_, c_3_, d_1_, d_2_, d_3_, e_1_, e_2_, f_1_, f_2_, h_1_, h_2_, h_3_, p_1_, p_2_, p_3_*, clearly discernible and directing backward (Figures 29, 30, 31).

Cuticular surface with elevated transversal thickenings (*tr.e.t*); *tr.e.t.1* with rounded promontories, situated in front of *c* setal alignment, externally to *c_1_* setae; smooth zone between *c*_1_ setal pair (Figure 29). Transverse bands: S2 clearly visible (Figures 29, 30, 31), situated behind *c* setal alignment. Thickenings *tr.e.t.2* and *tr.e.t.3* between *c* and *d* setal alignment; *tr.e.t.2* with rounded promontories, close to *c* alignment; *tr.e.t.3* smoothly surfaced, close to *d* setal alignment; longitudinal furrow running between *d_1_* setal insertions. Transverse band S3 observed between *tr.e.t.2* and *tr.e.t.3* (Figures 29, 30, 31). S4 situated posterior to *d* setal alignment. Transverse thickenings *tr.e.t.4* and *tr.e.t.5* between *d* and *e* setal alignment; *tr.e.t.4* smooth, with deep central furrow (Figure 30, indicated by¿) running along *tr.e.t4*; *tr.e.t5* with rounded promontories. S5 situated between *tr.e.t4* and *tr.e.t5*. U-shaped S6, with rounded promontories, observed between *e* and *f* setal alignment, situated on either side of *tr.e.t.6*. Posterior to *f* setal alignment, in oblique position, with central zone not corssing longitudinal medial plane, smooth *tr.e.t. 7*. S7 situated behind *tr.e.t.7*; *tr.e.t8* in oblique position, not crossing medial longitudinal plane, surface with rounded promontories. S8 behind *tr.e.t8*; *tr.e.t.9* in oblique position, not crossing medial longitudinal plane, smooth; S9 situated behind *tr.e.t.* A series of more or less triangular posterior promontories (*p.p*) observed in posterior medial zone (Figures 29, 30, 31). Only lyrifissure *ia* discernible anteriorly on frontal lobe of pleuraspis.

*Lateral region.* Bothridium (*bo*): margin elevated, ovoid, clearly visible (Figures 36, 38); *sb* depressed zone situated close to and behind *bo* and *in* (Figures 30, 31, 38); polyhedral microsculpture (Figure 45); small depressed marginal zone situated above longitudinal unsclerotized line (Figure 37). Rounded promontories easily visible (Figures 36, 38, 40). Prodorsal margin presenting conspicuous depression *l.d* (Figure 36), housing legs I–IV during leg folding. Polyhedral lobe with lyrifissure *ia* and rounded promontories (Figure 37) on anterior zone of pleuraspis. Conspicuous tectum on anterior notogastral zone. Unsclerotized longitudinal line easily discernible, exceeding level of *f_2_* setal insertions and clearly delimiting notaspis and pleuraspis (Figure 36).

*Ventral region.* Four pairs of subcapitular setae (Figure 49); setae *h*, *m_2_* and *a* clearly visible (more or less similar length); setae *m_1_* situated marginally and hardly discernible (Figures 32, 37, 49). Infracapitulum: complex microsculpture. Triangular microsculpture with rounded promontories in central zone between setae *h*, surrounded by smooth zone. Several areas with polyhedral microsculpture (Figure 49, indicated by ¿).

Epimeral zone: only epimere I with rounded promontories, easily observed in insertion zones of setae *1a, 1b, 1c* (Figure 47). Other epimeres smooth; epimeral setae variable on epimeres 3, 4 with formulae: 3–1–[3 (2)]–[4 (3)] (Figure 32 indicated by l). All setae similarly shaped (Figure 47). Genital plate undivided with nine to ten pairs of setae (Figures 32, 49); six or seven aligned paraxially and three or four antiaxially. Preanal plate typically shaped, characteristic of the genus (Figures 47–48). Anal plate fused with adanal, delimiting single plate with six pairs of setae (Figures 47, 48). BPAD clearly visible after lengthy soaking in lactic acid (Figure 32); lyrifissures *ia, ip, ih*, *ip* observed (Figures 32, 37).

*Legs.* Setal formulae I (0–4–3–3–16–1) (2–1–2); II (0–6–3–5–13–1 (1–1–1); III (2–2–3–3–13–1 (1–1–0); IV (2–3–3–2–13–1(1–0–0). See Table 2 and Figures 52–55.

###### Remarks.

Polyhedral microsculpture observed in several areas. Porous areas are very difficult to observe, as in most cases they are situated in the same zone as the microsculpture. On legs this microsculpture is present on all segments.

**Figures 42–47.**
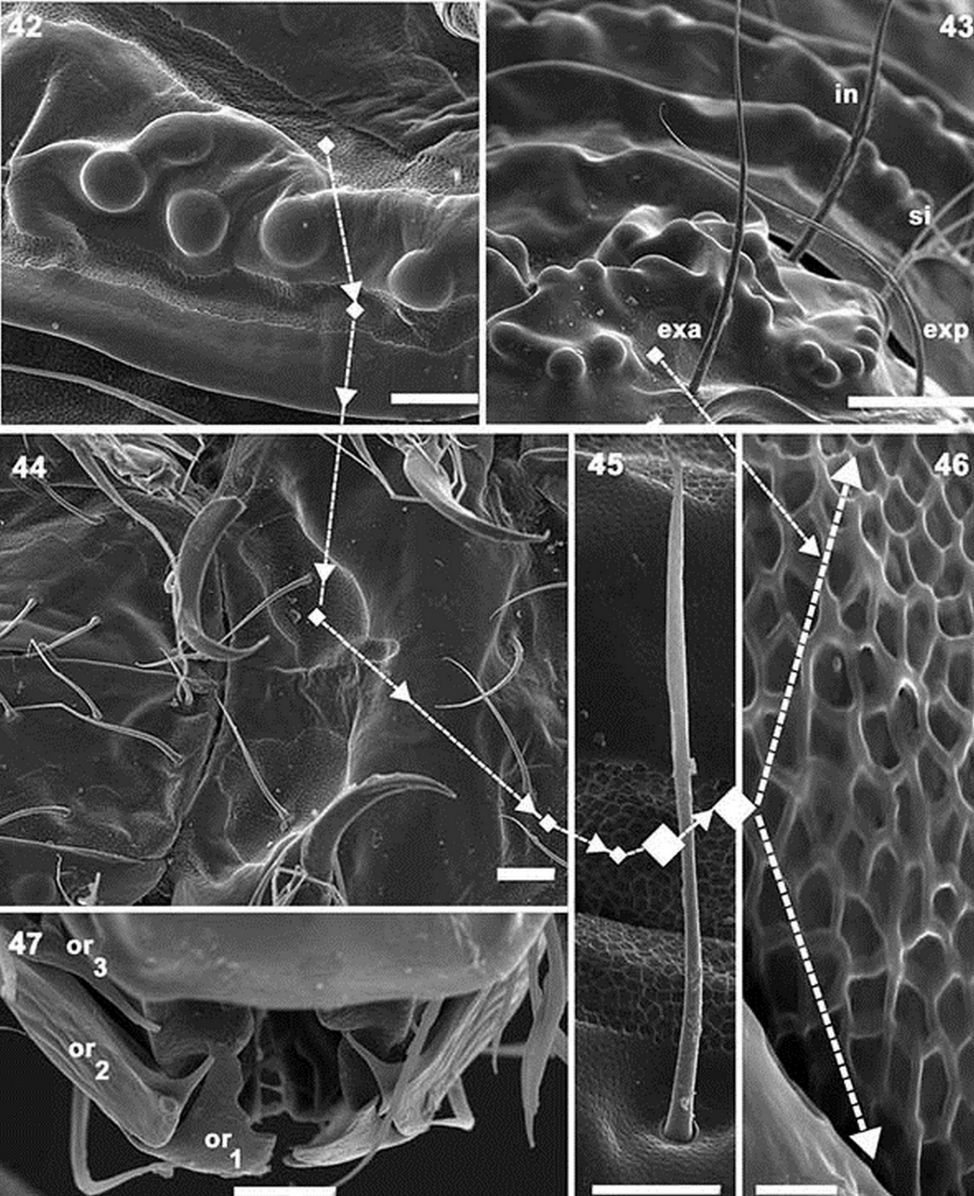
*Paulianacarus costaricensis* sp. n. Adult with cerotegumental layer. SEM. **42** lateral view, notogastral promontories **43** Frontal prodorsum detail promontories **44** epimeral posterior zone **45** notogastral setae and microsculpture, “porose area” **46** depressed area microsculpture, notogastral zone **47** adoral setae, frontal view. Abbreviations: See Material and methods. Scale bars: **42** = 20 μm; **43** = 50 μm; **44** = 20 μm; **45** = 10 μm; **46** = 2 μm; **47** = 10 μm.

**Figures 48–51.**
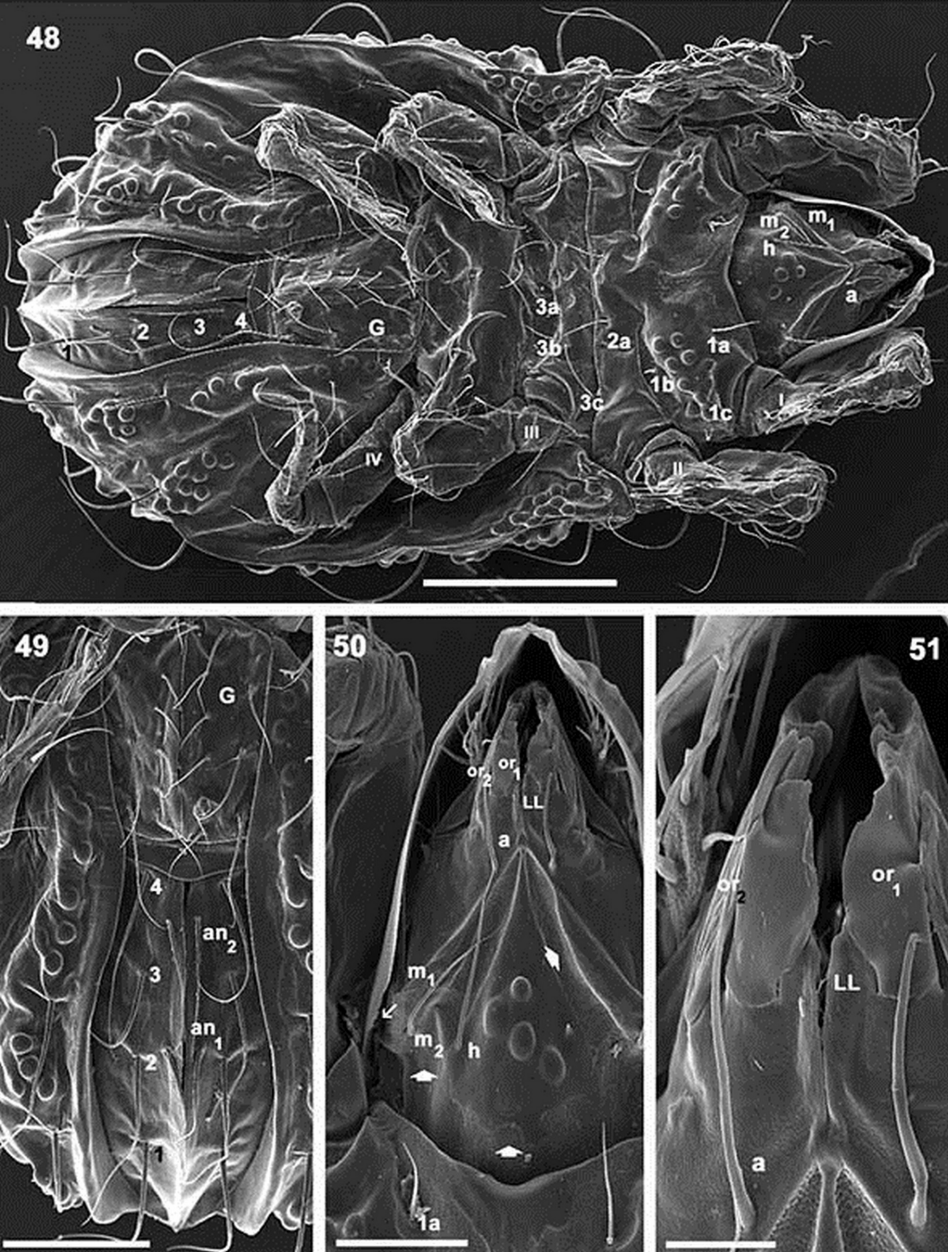
*Paulianacarus costaricensis* sp. n. Adult with cerotegumental layer. SEM. **48** ventral view **49** anogenital region **50** infracapitulum **51** apical infracapitular zone. Abbreviations: See Material and methods. Scale bars: **48** = 200 μm; **49** = 100 μm; **50** = 50μm; **51** = 20μm.

**Figures 52–55.**
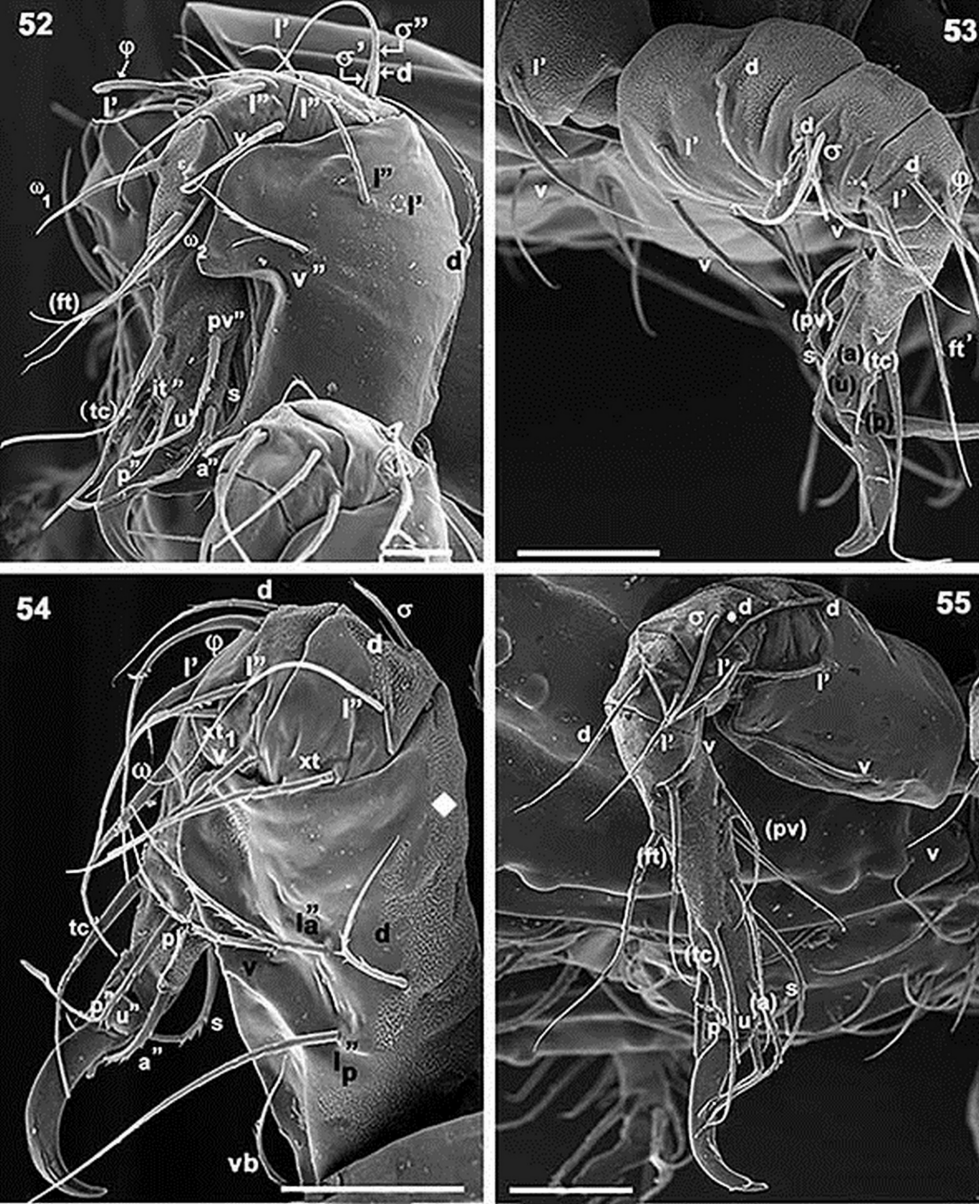
*Paulianacarus costaricensis* sp. n. Adult with cerotegumental layer. SEM. **52** leg I, antiaxial **53** leg III, antiaxial **54** leg II, antiaxial **55** leg IV, antiaxial. Abbreviations: See Material and methods. Scale bars: **52** = 20 μm; **53** = 50 μm; **54** = 50 μm; **55** = 50 μm.

## Discussion

The genus *Mixacarus* was proposed by Balogh (1958); but later Balogh and Balogh (1987) proposed another new genus, *Phyllolohmannia*. Currently *Mixacarus* is divided into two sub-genera, *Mixacarus* and *Phyllolohmannia*, and includes 22 species (Subias 2017).

A comparison between *Mixacarus turialbaiensis* sp. n. and *Mixacarus exilis* Aoki 1970 is quite complex, as some aspects of the initial description is detailed and others are deficient, for example: lateral observations are ignored and for the ventral region, drawings are referred to, but in a preceding paper by Wallwork 1962. Other problematic aspects include the absence of any reference to the porose areas of *M. exilis*. Making use of different study methods and technology, the authors were able to observe structures evidently not previously observed, such as the particular microsculpture in depressed areas (Figure 7), and transversal bands on notogaster (Figures 2, 5, 6, 13, 14, 16, 17, 19, 21, 23). Porose areas (Figure 8) were discernible on transversal notogastral band in the zone of this microsculpture.

*Mixacarus turialbaiensis* sp. n. is close to *Mixacarus exilis* Aoki 1970, but is differentiated by the depressed areas with particular microsculpture; all prodorsal setae have similar characteristics and length; ribbon-like bands on prodorsum distributed very differently; notogastral setae slightly barbate; nine transversal bands; epimeres with large number of depressed areas; variable chaetotaxy in genital and epimeral zone.

*Paulianacarus* was proposed by Balogh (1960) from Madagascar. Subias (2004) considered *Millotacarus* to be a subgenus of *Paulianacarus*. At present there are 15 species allocated to *Paulianacarus* and *Millotacarus*. The taxonomy of these genera are complex, and considering one a subgenus of the other is complicated by the lack of a detailed comparative study of type materials. Several authors have expressed their opinions (Mahunka 1985; Coetzee 2001; Chen et al. 2012, Fernandez et al. 2014), and these considerations highlight the incongruences in the descriptions, indicating that some do not consider that these are different genera, or do not consider one to be a subgenus of the other, while other researchers accept both subgenera. An analysis of these opinions is not repeated here in order to avoid redundancy. The only way to solve the problem is the study and comparison of type material, which was not possible in this instance.

The new species *Paulianacarus costaricensis* sp. n. was described using optical and SEM microscopy. These techniques allowed us to understand some of the complex structures also observed in *Paulianacarus rugosus* Balogh, 1961, a species close to the newly described species.

*P. costaricensis* displays the following characters: elevated transversal thickening (*tr.e.t*) with transverse bands: 1) some cross the transverse medial notogastral plane, others do not; 2) some are rectilinear, others oblique; 3) some present superficial rounded protuberances, others are smooth; 4) smooth thickenings either with complete furrow running the entire length, or partial furrow; 5) *tr.e.t* are associated with transversal furrows (*S*); 6) transversal furrows are related to one or both sides of the elevated transversal thickenings. Variable number and disposition of genital and epimeral setae, difficulty in observing lyrifissures. These are only some of the characteristics of this species, but they emphasize the need for detailed studies.

*P. rugosus* Balogh 1961 is close to *P. costaricensis*, but is differentiated by the presence of prodorsal transversal band; barbate *in* setae; elevated transversal thickening, transverse medial notogastral plane *tr.e.t* 1, 2, 3, 4, 5; *tr.e.t* 6, 7, 8, 9 not crossing medial notogastral plane; elevated transversal thickening with rounded protuberances: *tr.e.t* 1, 2 ,5, 6, 8, 9; with smooth surface: *tr.e.t* 3, 4, 7; epimeral chaetotaxy: (3–1–[3 (2)]–[4 (3)]); porose area rounded, very difficult to observe as it is situated in polyhedral microsculpture zone; genital setae variable with 9–10 pairs, of which 6–7 are aligned paraxially and 3-4 antiaxially.

## Supplementary Material

XML Treatment for Mixacarus turialbaiensis

XML Treatment for Paulianacarus costaricensis
